# Complement mediators: key regulators of airway tissue remodeling in asthma

**DOI:** 10.1186/s12967-015-0565-2

**Published:** 2015-08-20

**Authors:** Mohammad Afzal Khan, Abdullah Mohammed Assiri, Dieter Clemens Broering

**Affiliations:** Department of Comparative Medicine, King Faisal Specialist Hospital & Research Centre, P.O. Box 3354, Riyadh, 11211 MBC-03 Kingdom of Saudi Arabia; Organ Transplant Centre, King Faisal Specialist Hospital & Research Centre, Riyadh, Kingdom of Saudi Arabia

**Keywords:** Complement mediators, Airway remodeling, Anaphylatoxins

## Abstract

The complement mediators are the major effectors of the immune balance, which operates at the interface between the innate and adaptive immunity, and is vital for many immunoregulatory functions. Activation of the complement cascade through the classical, alternative or lectin pathways thus generating opsonins like C3b and C5b, anaphylatoxins C3a and C5a, chemotaxin, and inflammatory mediators, which leads to cellular death. Complement mediators that accelerate the airway remodeling are not well defined; however, an uncontrolled Th2-driven adaptive immune response has been linked to the major pathophysiologic features of asthma, including bronchoconstriction, airway hyperresponsiveness, and airway inflammation. The mechanisms leading to complement mediated airway tissue remodeling, and the effect of therapy on preventing and/or reversing it are not clearly understood. This review highlights complement-mediated inflammation, and the mechanism through it triggers the airway tissue injury and remodeling in the airway epithelium that could serve as potential targets for developing a new drug to rescue the asthma patients.

## Introduction

**Fig. 1:**
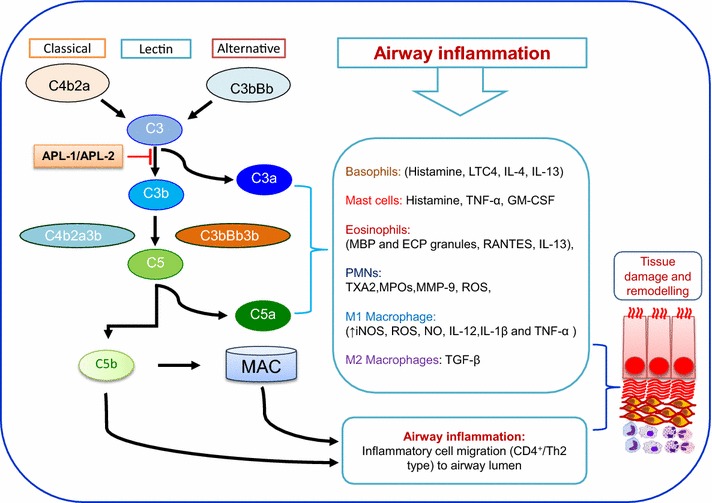
Model shows active mediators of the complement cascade during inflammation in airway asthma. Further, it highlights activation of inflammatory cells (Basophils, PMNs and Macrophages) through complement receptor binding and downstream release of inflammatory mediators. As shown, all these mediators contribute in tissue damage and remodeling. APL-1 and APL-2, are derivatives of Compstatin, bind to and inhibit complement activation at the C3 level, thus blocking all major effector pathways of complement activation. (Both APL-1 and APL-2 are under clinical trials).

**Fig. 2:**
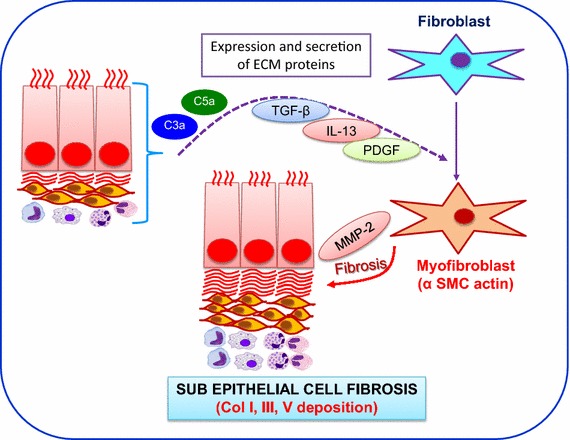
Model shows IL-13 and TGF-β mediated sub-epithelial fibrosis. Both of these cytokines are released post complement activation in airway lumen. Further, TGF- β1 promotes the differentiation of fibroblast into myofibroblast, and IL-13 stimulates collagen type-1 production by the airway fibroblast in a matrix metalloproteinase (MMP)-2 and TGF-β1-dependent manner.

Asthma is a chronic inflammatory disease of the airways distinguished by the variable airflow obstruction and associated increase in airway hyperresponsiveness (AHR) to various stimuli [1–3]. Asthma is considered to be mediated primarily by the allergen-specific CD4^+^ T cells, Th2 cytokines, and allergen-specific IgE antibody leading to airway inflammation and hyperresponsiveness [4–7]. Cellular inflammation of the diseased airways with eosinophil and neutrophil is the hallmark feature of asthma and is considered relevant to the pathogenesis of the disease [8–10]. Asthma pathogenesis has been associated with major pathophysiologic features including airway constriction, hyperresponsiveness, and neutrophilic inflammation [11–14] (Figs. 1, 2). In addition, over-activated complement cascade play a key role as effectors of cell-mediated and humoral immune system in pulmonary tissue injury during asthma pathogenesis [15–17].

In the recent years, a novel research area has centered on the role of innate immune components-complement cascade in the regulation of Th2-biased adaptive immunity in asthma [14, 18]. It is well established that uncontrolled complement activation in the airway contributes to asthma pathogenesis, which includes morphogenetic/or pulmonary tissue remodeling [19, 20]. Complement activation has been demonstrated in mouse models of allergic asthma, which highlighted the role of C3a mediated airway hyperresponsiveness, and airway tissue remodeling [21]. The complement system has also been associated with a variety of non-immunological conditions including pulmonary tissue regeneration, and progression to pulmonary tissue fibrosis after airway tissue injury [22]. The presence of increased levels of C3a and C5a peptides in the bronchoalveolar lavage (BAL) and serum of asthmatics and the increased expression of their respective receptors signifies the key involvement of the complement mediators in asthma pathogenesis [23]. In addition, it has been noted that levels of C reactive protein (CRP) are increased in non-allergic but not in allergic asthma conditions [24]. CRP is an acute-phase serum protein and a mediator of innate immunity through the complement activation of C1, C4, C2 and C3 components. CRP mediates innate immunity through the binding to microbial polysaccharides and to ligands exposed on damaged cells leading to the activation of the classical complement pathway, which facilitates uptake by phagocytic cells [25, 26].

The complement cascade comprises a network of active complement mediators of more than 30 proteins, which have been recognized as a major defense option for a host cell against the microbial invaders [19, 27] (Fig. 1). The complement cascade can be initiated through three different pathways: the classical pathway, the lectin pathway, and the alternative pathway. The alternative pathway of the complement cascade plays a vital role in neutrophil-mediated diseases including asthma [28, 29]. The three pathways all converge in the activation of the pivotal complement molecule C3 and generate C3 convertase. This C3 convertase facilitates the cleavage of C3 into C3a and C3b. After cleavage of C3, the C3b molecule combines with the C3 convertase to form a C4bC2aC3b complex in the classical and lectin pathways, while forming a C3bBbC3b complex in the alternative pathway. Both the C4bC2aC3b and C3bBbC3b complexes are known as C5 convertase, which catalyzes the cleavage of C5 into C5a and C5b molecules. The generated C5a can then act as a potent anaphylatoxin at the site of production while C5b participate in the assembly of the membrane attack complex (C5b-9 or MAC). The primary cytolytic activity of complement is catalyzed by the non-enzymatic function of the MAC, which is initiated through the amphiphilic complex generated after the linkage of C5b–C6, and C7. This complex is able to insert into microbial lipid bilayers and subsequent binding of C8 initiates C9 polymerization, which creates membrane-spanning channels to initiate osmotic lysis of the foreign constructs [19] (Fig. 1). In summary, the activated complement mediators in asthma airway tissue remodeling are recognized as a potential targets for therapeutic intervention in preclinical and clinical research [30–32]. This review primarily focus on indirect (thorough adaptive immune system) and direct effects of the complement system on airway tissue remodeling.

## Complement system effect on airway tissue remodeling mediated by adaptive immune system

The complement activation modulates key adoptive immune responses, which stimulates and/or suppress pulmonary allergic reactions during airway tissue remodeling [33]. The complement mediators exerts their immunoregulatory roles through the initiation and development of the adaptive immunity to stimulate and/or suppress pulmonary allergy in allergic asthma. The involvement of C3a and C5a in asthma is well established, and it is reported that both bronchial epithelial and smooth muscle cells express receptors for C3a and C5a anaphylatoxins [16, 23]. The core portion of complement activation cascade is involved in host defense against pathogenic infections; however, the by-products C3a and C5a of the pathway have potent inflammatory properties [15, 34]. In particular, complement components, and their activated fragments C3a and C5a synchronize the magnitude of adaptive immune responses via ligation of their respective receptors expressed on antigen-presenting cells (APCs) and T lymphocytes [1, 35–37]. A gradient of inflammatory cytokines released by Th2 cells particularly IL-4, IL-5 and IL-13 coordinates major pulmonary inflammatory responses, which involves development, migration, inflammatory cells activation, allergen-specific antibody production, goblet cell hyperplasia, increase in vascular permeability, and airway reactivity, all of which plays a central role in asthma pathogenesis, and pathology [4, 14, 38]. The genetic interference of complement components in asthma pathogenesis has been investigated, and it is reported that genetic variations in the complement components modulates the susceptibility to asthma [39]. A detailed single nucleotide polymorphisms (SNPs) in C3, C5, C3AR1, and C5AR1 gene plays a significant role in susceptibility and pathogenesis of bronchial asthma [1]. In addition, C3a and C5a modulates Th2 and Th17 immunity as the expression of IL17 by Th17 cell enhances C3 secretion by airway epithelial cells in severe allergic asthma [40].

The anaphylatoxins C5a and C3a have long been credited as a potent pro-inflammatory mediators contributing to the allergic inflammatory conditions [35]. Recent studies indicate that C3a regulate interaction of APCs and effector cells causing leukocyte activation, smooth muscle contraction, increase in vascular permeability [35, 41], inflammatory cell infiltration and mucus secretion [2, 3, 16].

A wide range of inflammatory cells have been associated with airway tissue remodeling includes lymphocytes, neutrophils, eosinophils, and macrophages, which releases various mediators to facilitates airway tissue remodeling process. A variety of cell type produces both mediators, however several studies have identified macrophages as a critical source of both TGF-β1 and PDGF in airway fibrosis [42, 43]. Notably, TGF-β is one of the leading mediator involved in airway tissue remodeling during asthmatic pathogenesis. This profibrotic cytokine is secreted by different inflammatory and epithelial cells including ASM, endothelial cells, fibroblasts, macrophages, and eosinophils [44]. TGF-β is a potent regulator of fibroblast/myofibroblast function and the production of several ECM proteins including collagens, proteoglycans, and tenascin [44, 45]. TGF-β1 is well known as a key inducer of fibrosis in many tissues and organs [46]. In addition, macrophage also generate profibrotic mediator especially platelet-derived growth factor (PDGF), which directly activate fibroblasts [44, 47]. PDGF act as a potent pro-fibrotic signal by stimulating the proliferation of activated collagen-producing hematopoietic stem cells (HSCs).

## Local effects of the complement system on the airway tissue remodeling

Airway tissue remodeling is a secondary process, which occur due to a chronic inflammatory environment [48, 49]. It disrupts the normal airway tissue repair process, which involves airway wall thickening, mucous hyperplasia, mucosal neovascularization, smooth muscle hyperplasia, deposition of fibrous and other extracellular matrix protein, myofibroblast hyperplasia, epithelial hypertrophy, and mucous gland and goblet cell hyperplasia [48]. Although, the connection between inflammatory process and fibrotic remodeling is well recognized but the origin, mechanism of activation, differentiation of fibroblasts, and the role of different inflammatory cells in asthma is still remain unclear. The relation between complement system and asthma pathogenesis has been previously investigated in different animal model systems [17, 50, 51]. The complement system, with its crucial role in innate and adaptive immunity mediates a variety of effector functions [3, 32, 41] that play a key roles in airway tissue inflammation and injury. It is a complex cascade involving proteolytic cleavage of anaphylatoxins (C3a and C5a), which are often activated by cell receptors. This cascade ultimately leads to the generation of antibodies and inflammatory responses as well as opsonization of apoptotic and necrotic bodies, facilitating their recognition, clearance, and lysis [20, 27]. C3a and C5a trigger ASM contraction, promote mucus secretion, and augment blood vascular permeability [20, 35]. Furthermore, C5a and its end-product, membrane attack complex (MAC) regulate the downstream inflammatory responses through the infiltration of inflammatory cells into bronchial airway lumen, which stimulate the release of multiple acute inflammatory mediators TGF-β, RANTES and Pro MMP-9 [52, 53]. These inflammatory mediators may induce ASM hypertrophy and collagen deposition under the respiratory epithelium, which leads to the airway tissue remodeling and repair of lower airways [54].

C3a and C5a have potential to activates inflammatory immune cells such as mast cells, macrophages, neutrophils, basophils, eosinophils, and also facilitates enhanced vascular permeability (through bradykinin), and triggers smooth muscle contraction through their receptors [55]. In allergic diseases, C3a and C5a mediated C3aR and C5aR stimulation respectively produces a series of effector functions ranging from inflammatory cell migration to pro-inflammatory mediator production thus contributing to the development of airway remodeling [56]. Complement receptor-mediated activation of mast cells can occur through CR3 (the receptor for C3b) and C3aR (receptors for C3a); however, degranulation seems to occur mainly as a result of activation of the receptor for C5a (C5aR) [57, 58]. Activated mast cells secrete TNF-α, IL-3, and GM-CSF, which facilitates downstream activation of neutrophils, eosinophils, and basophils respectively (Fig. 1; Table 1). During eosinophil activation, C3a and C5a regulate the production of eosinophil cationic protein and their adhesion to endothelial cells as well as their migration [59]. C3a mediate synthesis of IL-6 and TNF-α from B cells and monocytes, and IL-17A from Th17 cells, which control the severity of experimental asthma [40, 60, 61]. The anaphylatoxins C3a and C5a generated during complement activation holds numerous pro-inflammatory and immunoregulatory properties critical for development and modulation of allergic immune responses [62]. In addition to its pro-inflammatory effector functions, complement regulates adaptive immunity at many levels [4, 63] including increased neutrophilic inflammation in asthma [64].

**Table 1:** Signaling through complement mediators and immune cells in airway tissue remodeling

Complement	Immune cells	Signaling molecule	Remodeling
C3a, C5a	M2 macrophages	TGF-β	Expression and secretion of ECM proteins
C3a, C5a	M1 macrophages	↑iNOS, ROS, NO, IL-12, IL-1β and TNF-α	AHR, airway fibrosis, attraction of eosinophils and neutrophils
C3a, C5a	Eosinophil	MBP and ECP granules, RANTES, IL-13, LTC4, LTC4 and LTE4, TGF-β	Vascular permeability, mucus secretion, and ASM contraction, modulation of cellular trafficking
C3a, C5a	Basophils	Histamine, LTC4, IL-4, IL-13	ASM contraction, vascular permeability, promotes Th2 and IgE production
C3a, C5a	PMNs	TXA2, MPOs, MMP-9, ROS	Bronchoconstriction, stimulate release of serotonin and histamine through platelets and mast cells respectively. Vascular permeability, mucus hypersecretion
C3a, C5a	Mast cells	Histamine, TNF-α, GM-CSF, IL-4, IL-13 LTC4, LTB4 and PGD2	Stimulates ASM contraction, vasodilatation and release of IL-16 production by CD8+ cells and airway epithelial cells
C5b and MAC	Th2/CD4+	IL-4, IL-5 and IL-13	IL-13 suppress activation of NF-kB, and concomitant IL-5 induced eosinophilic inflammation in an IL-4- independent manner

C5a is a potent chemoattractant for macrophages [65], neutrophils [66], activated B [67] and T cells [68], basophils [69] and mast cells [70]. Complement-induced neutrophil activation mostly involve C5a and possibly C5b-9 complexes identified in serum-activated neutrophils [71]. The products of eosinophil activation, and bindings of C3a and C5a have been reported to cause mast cell degranulation in lung tissues, which further regulates the process of inflammation and remodeling [72] (Fig. 2). In addition, C5a has been shown as one of the mediator of pulmonary fibrosis through the release of TGF-β and IL-13 by M2 macrophage and eosinophils respectively [70, 73, 74].

The anaphylatoxins C3a and C5a produced during complement cascade activation influence numerous pro-inflammatory and immunoregulatory mediators for the pulmonary tissue-specific immune responses [36] (Table 1). Both C3a and C5a have ability to recruit leukocytic effector cells of the allergic inflammatory response [20]. Of note, C5a is a potent chemo-attractant for cells like macrophages, basophils, neutrophils, and T lymphocytes, whereas both anaphylatoxins can be chemotactic for eosinophils and mast cells [11]. C3a and C5a also have potency to activate infiltrating granulocytes, leading to speedy production and release of pro-inflammatory mediators, such as histamines, leukotrienes, and platelet-activating factor as well as pro-inflammatory cytokines and chemokines, including IL-1, IL-6 and TNF-α [75–78]. The generation of functional complement mediators occurs in asthmatic persons, and both C3a and C5a are essential contributors in the pathophysiology of the disease [20]. In asthmatic individuals, a rise in serum C3a and C5a have been reported after allergen-induced bronchospasm [79]. Additionally, the levels of C3a and C5a have been found to increase in the BAL of asthmatic patients after segmental allergen challenge [16]. One study found that C3a and C4a concentrations were elevated in the plasma of patients with aspirin-induced asthma [80], and numerous other clinical studies has also reported the significant production of anaphylatoxins under asthmatic conditions [18]. However, studies on animal models of airway hyperresponsiveness (AHR) has also concluded that both complement C3a and C5a are critical for asthma pathogenesis [16, 23, 81]. In addition to allergen-mediated activation, environmental agents can also triggers the complement activation. Airborne pollutants/or airborne particulate matter can activate the alternative pathway of the complement cascade in human serum and airway epithelium, respectively [34]. Cigarette smoke (CS) has been shown to activate the alternative pathway through cleavage of the internal thio-ester bond in C3 [82]. C3a also stimulates smooth muscle contraction [83], lysozyme release from immune cells [84], platelet aggregation [85], and triglyceride synthesis in adipocytes [86].

## Discussion

Airway tissue remodeling in asthma is pathologically characterized by subepithelial deposition of collagen in the airways, increase in ASM cell mass, mucus gland hyperplasia, and mucosal neovascularization [48, 87–90]. The process of airway remodeling is potentially a crucial outcome of asthma, and has been associated with the increase in inflammatory cells specially macrophages, neutrophils, basophils and their mediators TGF-β, iNOS, ROS, NO, IL-12, IL-1β, TNF-α, TXA2, MPOs, MMP-9, ROS, histamine, LTC4, IL-4, and IL-13, which affect airway structural property and pulmonary functions [47, 74, 91, 92]. Thus, it is most likely that the process of airway tissue remodeling precede to physiologic subphenotypes of irreversible/or partially reversible airflow obstruction and accelerated lung functions decline associated with the severity of disease [49].

**Fig. 3:**
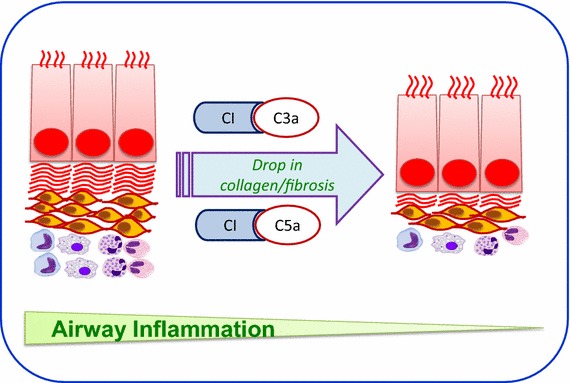
Simple illustration of complement inhibition approach to prevent fibrosis. This *figure* shows how blocking C3a and C5a prevent downstream activation of inflammatory mediators and subdue fibrotic process.

Therapeutically, complement mediators C3a and C5a, and their respective receptors C3aR and C5aR, displays diverse activities during the course of disease progression and drugs that specifically targets C3a, C3aR, C5, C5a or C5aR could serve as potential therapeutic options for asthma treatment in future (Fig. 3) [32]. Experimental findings in mouse model of chronic allergen challenge have demonstrated some of the interesting correlations between airway inflammation, dysfunction and tissue remodeling [93]. The specific roles of complement fragments have been reported in other disease models, e.g. systemic inhibition of C3a and C5a resulted in down regulation of collagen have been reported in a mouse model of orthotopic tracheal transplantations [22, 94]. It has been reported that C5-knockouts and C5aR antagonism significantly reduced airway tissue fibrosis at 5 and 10 days post-injury in a mouse model of unilateral urethral obstruction [95]. These investigations in animal models will increasingly allow categorizations of potential mediators resulting in airway tissue remodeling, and their impact on airway physiology [3, 32, 96]. In addition, new complement inhibition based drugs are now under development and shown significant attention in pulmonary diseases in human. In clinical trials, Compstatin, and its derivatives APL-1 and APL-2 bind to and inhibit complement activation at C3 activation step, thus blocking all major effector pathways required for complement mediated tissue injury (Fig. 1). C3 inhibition has the potential to make available a broad and effective treatment comparable to further complement inhibition approaches. APL-1, is as a disease-modifying therapy for severe asthma and chronic obstructive pulmonary disease and an inhalable formulation of APL-1 is being used in ongoing Phase 1 trial in the United Kingdom [97, 98]. These studies will rectify efficacy of asthma treatments in reducing airway tissue remodeling in asthma patients. However, additional efforts are necessary in order to uncover the relationship between changes in airway pathology, and physiology before treatment regimens to prevent/or resolve established tissue airway remodeling. In new drug development for asthma therapy, the overwhelming interest of various highly specific complement inhibitors (C3a and C5a) for the demonstration of pathological mechanisms may not only uncover new candidates with therapeutic potential but also help discover even more fascinating cross-talk mechanisms between complement and other cellular parts of immunity.

In summary, this review discussed the complement-mediated airway tissue injury, remodeling in airway epithelium, and we anticipate that blocking/or antagonism of the functioning complement mediators could act as a potential therapeutic strategy to salvage asthma patients.

## Conclusions

This review contributes to distinguish direct effects of the complement system on biological processes associated with airway tissue remodeling from indirect effects caused by adaptive immunity. Of note, this review highlights that complex functional changes in airways coexist with the complex inflammatory processes, and the potential synergistic use of C3a and C5a inhibition may subdue airway inflammation and prevent subepithelial fibrosis by blocking the intrapulmonary activation of C3a and C5a, is a potential clinical approach for treating patients with asthma.

## Abbreviations

APL-1 and -2: novel complement inhibitors of Apellis pharma
AHR: airway hyperresponsiveness
ASM: airway smooth muscle
BAL: bronchoalveolar lavage
C3AR1: complement component 3a receptor 1
C5AR1: complement component 5a receptor 1
CI: complement inhibitors
CRP: C reactive protein
CS: cigarette smoke
ECM: extra cellular matrix
ECP: eosinophil cationic protein
HSCs: hematopoietic stem cells
MPOs: myeloperoxidase
NO: nitric oxide
iNOS: inducible nitric oxide synthetase
PDGF: platelet-derived growth factor
PGD2: prostaglandin D2
Pro MMP-9: matrix metallopeptidase 9 (Type IV collagenase)
RANTES: regulated on activation, normal T cell expressed and secreted
ROS: reactive oxygen species
TXA2: thromboxane A2
